# Topoisomerase IIbeta is required for proper retinal development and survival of postmitotic cells

**DOI:** 10.1242/bio.20146767

**Published:** 2014-01-17

**Authors:** Ying Li, Hailing Hao, Evangeline Tzatzalos, Ren-Kuo Lin, Sungtae Doh, Leroy F. Liu, Yi Lisa Lyu, Li Cai

**Affiliations:** 1 Department of Biomedical Engineering, Rutgers University, 599 Taylor Road, Piscataway, NJ 08854, USA; 2 Department of Pharmacology, Rutgers University-Robert Wood Johnson Medical School, 675 Hoes Lane, Piscataway, NJ 08854, USA

**Keywords:** Cell survival, Gene regulation, Postmitotic cells, Retina, Top2b, Transcription, Retina degeneration

## Abstract

Topoisomerase IIbeta (Top2b) is an enzyme that modulates DNA supercoiling by catalyzing the passage of DNA duplexes through one another. It is ubiquitously expressed in postmitotic cells and known to function during the development of neuromuscular junctions in the diaphragm and the proper formation of laminar structure in the cerebral cortex. However, due to the perinatal death phenotype of the traditional constitutive and brain-specific Top2b knockout mice, the precise *in vivo* function of Top2b, especially during postnatal neural development, remains to be determined. Using both the constitutive and retina-specific knockout mouse models, we showed that Top2b deficiency resulted in delayed neuronal differentiation, degeneration of the plexiform layers and outer segment of photoreceptors, as well as dramatic reduction in cell number in the retina. Genome-wide transcriptome analysis by RNA sequencing revealed that genes involved in neuronal survival and neural system development were preferentially affected in Top2b-deficient retinas. Collectively, our findings have indicated an important function of Top2b in proper development and the maintenance/survival of postmitotic neurons in the retina.

## Introduction

Type II DNA topoisomerases (topoisomerase II, Top2) are DNA machines that are capable of catalyzing an ATP-dependent passage of one DNA duplex through another (Wang, 1998). This activity is essential in removing unconstrained DNA supercoiling during various DNA transactions (Wang, 2002; Nitiss, 2009). In mammals, two evolutionally conserved Top2 isozymes (Top2a and Top2b) encoded by separate genes are present; and they possess similar *in vitro* catalytic activities (Wang, 2002; Nitiss, 2009). Top2a is expressed solely in proliferating cells, whereas Top2b is ubiquitously expressed in terminally differentiated cells including neurons and cardiomyocytes (Tsutsui et al., 1993; Lyu and Wang, 2003; Zhang et al., 2012). While Top2a is essential in proliferating cells and has been linked to DNA replication and chromosome condensation/segregation, Top2b has been clearly indicated in regulating gene expression (e.g. Reln, Dab1, Catna2, Cdh13, Sst, Pbx3, and Epha7) during brain development (Lyu and Wang, 2003; Lyu et al., 2006; Nur-E-Kamal et al., 2007) and in facilitating transcription of autism spectrum disorder-linked genes (King et al., 2013).

In the developing mouse cerebral cortex, Top2b is absent from proliferating neural progenitors located in the ventricular zone and subventricular zone, but expressed in postmitotic neurons undergoing terminal differentiation in the cortical plate region (Lyu and Wang, 2003). A similar pattern of Top2b expression has also been observed in other regions of the central nervous system (CNS), e.g. the cerebellum (Tsutsui et al., 1993; Tsutsui et al., 2001a; Tsutsui et al., 2001b). Ablating Top2b in mice leads to neural developmental defects such as defective innervation of motor neurons in the diaphragm muscle (Yang et al., 2000), abnormal migration of cerebral cortical neurons, and aberrant lamination of the cerebral cortex (Yang et al., 2000; Lyu and Wang, 2003). In addition, Top2b is required for proper neurite outgrowth and axon path-finding (Nur-E-Kamal et al., 2007; Nevin et al., 2011). These findings indicate the importance of Top2b in neural development. Indeed, it has been shown that Top2b controls the expression of many developmentally regulated genes (e.g. Reln, Dab1, Epha gene family) during mouse embryonic brain development (Lyu et al., 2006), as well as gene activation in rat cerebellar granule cells (Tsutsui et al., 2001a; Sano et al., 2008). Furthermore, although Top2b is apparently nonessential in cultured cells, absence of Top2b in embryonic stem cell (ESC)-derived neurons results in premature cell death (Tiwari et al., 2012). However, *in vivo* evidence supporting an essential role of Top2b in the survival/maintenance of postmitotic neurons is lacking.

To study the *in vivo* function of Top2b in postmitotic neurons, we have previously generated brain-specific Top2b knockout (KO) mice by breeding floxed Top2b mice (Lyu and Wang, 2003) with Foxg1-Cre mice (Hébert and McConnell, 2000). Unfortunately, these mice showed a perinatal death phenotype, similar to that observed in the traditional constitutive Top2b KO mice (Yang et al., 2000; Lyu and Wang, 2003). To circumvent this perinatal death problem, we have employed the developing mouse retina as a model to further analyze the *in vivo* function of Top2b. Retina is not essential for animal survival, and as a part of the CNS, it provides an excellent model for the study of neural development and pathogenesis. In vertebrate retina, there are six types of neurons and one type of glia interconnecting with one another to form a sophisticated neuron/glia network that relays visual input into the brain. The mature vertebrate retina is organized in a laminar structure composed of three cellular layers and two plexiform layers (basal to apical): ganglion cell layer (GCL), inner plexiform layer (IPL), inner nuclear layer (INL), outer plexiform layer (OPL) and outer nuclear layer (ONL). The genesis of mouse retinal cell types proceeds through an overlapping and yet temporal-controlled order: ganglion cells are born first around embryonic day 10 (E10), followed by cone photoreceptors, horizontal cells and amacrine cells at around E13∼E15, whereas the majority of rod photoreceptors, bipolar neurons, and Müller cells are generated after birth (Young, 1985b; Young, 1985a; Bassett and Wallace, 2012).

In this study, by employing both the traditional constitutive Top2b KO (KO) and retina-specific conditional Top2b KO (cKO) mouse models, we show that the initial specification of retinal progenitors into different retinal cell lineages was not affected by Top2b deficiency. However, retina lacking Top2b displays defects in the laminar structure and neurite outgrowth. In addition, Top2b deficiency led to a decrease in retinal thickness and an increase in apoptotic cell depth at later developmental stages. These results suggest a link between Top2b deficiency and retinal neurodegeneration and imply an essential role of Top2b in maintaining the function and survival of postmitotic neurons. Genome-wide transcriptome analysis of Top2b-deficient retinas using the RNA-seq method further confirms that Top2b regulates gene networks critical for neuronal survival and neurite outgrowth. Together, these results suggest that although it may have a minimal effect on retinal lineage specification, Top2b is vital in maintaining the postmitotic state and survival of retinal neurons.

## Results

### Top2b expression is only present in postmitotic cells during mouse retinal development

**Fig. 1.**
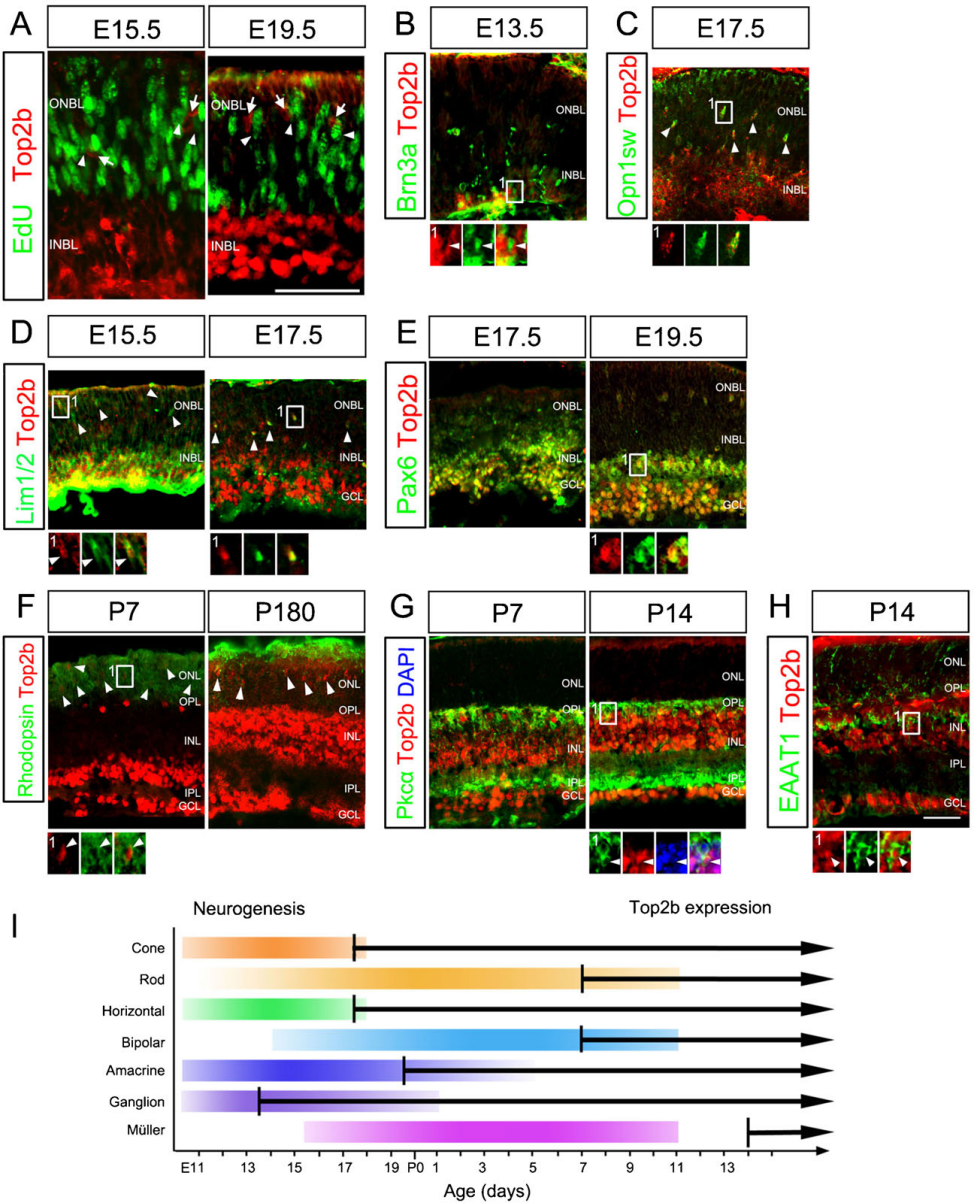
Expression pattern of Top2b during mouse retinal development. (A) E15.5 and E19.5 retina sections were processed to reveal EdU *in vivo* labeling. Staining with Top2b show that the vast majority of Top2b+ cells were in the INBL while EdU+ cells were in the ONBL. No EdU+ cells (arrowheads) were co-labeled with Top2b (arrows). (B) Top2b+ cells were co-labeled with Brn3a at E13.5. (C) Top2b+ cells were co-labeled with Opn1sw at E17.5. (D) Co-staining of Lim1/2 and Top2b at E15.5 and E17.5. (E) Co-staining of Pax6 and Top2b at 17.5 and E19.5. (F) Co-staining of Rhodopsin and Top2b at P7 and P180. (G) Co-staining of Pkcα and Top2b at P7 and P14. (H) Co-staining of EAAT1 and Top2b at P14. Boxed regions are shown in a higher magnification. Arrowheads in (B–H) indicate double-labeled cells with Top2b and a retinal cell type specific marker. (I) Timeline of Top2b expression and neurogenesis in mouse retina. Color bars indicate the process of retinal development based on Young's work (Young, 1985a). Black arrows represent the timing of Top2b expression in different retinal cell types. INBL, inner neuroblastic layer; ONBL, outer neuroblastic later; GCL, ganglion cell layer; INL, inner nuclear layer; IPL, inner plexiform layer; ONL, outer nuclear layer; OPL, outer plexiform layer. Scale bars: 50 µm.

To determine whether Top2b is expressed in proliferating or postmitotic cells, we pulse-labeled proliferating retinal progenitors with the thymidine analog EdU (5-ethynyl-2′-deoxyuridine) (Salic and Mitchison, 2008) and immunostained retinal sections with Top2b antibody at embryonic day 15.5 (E15.5) and E19.5. We found that no cells were co-labeled with Top2b and EdU in the retina (Fig. 1A), indicating that Top2b was not expressed in proliferating retinal progenitors.

Next, we determined the onset of Top2b expression in each specific retinal cell type by double immunostaining for Top2b and cell-specific markers, and by cell-specific laminar location in the retina. Top2b expression was not detected before or at E12.5 (data not shown). Weak Top2b staining was detected first in regions near the vitreous surface of the inner neuroblastic layer (INBL) at E13.5 (Fig. 1B; supplementary material Fig. S1A). Many Top2b+ cells were co-labeled with Brn3a, a differentiated ganglion cell marker (Xiang et al., 1995; Xiang, 1998) (Fig. 1B). Starting from E17.5, Top2b expression became apparent in newly born Opn1sw+ cone photoreceptors (Fig. 1C), Lim1/2+ horizontal cells (Fig. 1D), and Pax6+ amacrine cells (Fig. 1E). In postnatal stages, Top2b expression was maintained in postmitotic cells of both neuronal and glial origin, including Rhodopsin+ rod photoreceptors (Fig. 1F), Pkcα+ bipolar cells (Fig. 1G), and EAAT1+ Müller glia (Fig. 1H), subsequent to their differentiation.

The expression pattern of Top2b in retinal cells correlated well with the period of retinal neurogenesis and overlapped with the period of retinal cell differentiation (Fig. 1I; supplementary material Fig. S1). These results indicate that Top2b is expressed and maintained in all differentiated and mature retinal cell types.

### Top2b deficiency does not affect early mouse embryonic neurogenesis but causes morphological defects in the postnatal eye

The role of Top2b during embryonic retinal development was examined using the traditional constitutive Top2b knockout (KO) embryos. Immunostaining showed no Top2b expression in the KO retinas (supplementary material Fig. S2A,B) and no discernible differences in staining with retinal ganglion cell markers Brn3 (detects Brn3a, Brn3b and Brn3c for both ganglion progenitors and differentiated ganglion cells (Xiang et al., 1993; Xiang, 1998)) and Brn3a, cone photoreceptor marker Opn1sw, retinal progenitor marker Pax6 (Marquardt et al., 2001) and early neuronal marker Tuj-1 at E13.5 and E15.5 (supplementary material Fig. S3). In addition, there is no obvious difference in the number of EdU+ cells and phosphorylated-histone 3 (PH3)-labeled M-phase cells between wild-type and Top2b KO retinas (supplementary material Fig. S4). These results suggest that early retinogenesis and retinal progenitor proliferation do not require Top2b.

Since both the constitutive (Yang et al., 2000) and brain-specific Top2b KO mice die shortly after birth (Lyu and Wang, 2003), these models cannot be used to study the role of Top2b during the postnatal CNS development. To circumvent this problem, a retina-specific Top2b KO (cKO) mouse line was generated by crossing the floxed Top2b mice (Lyu and Wang, 2003) with the *Dkk3-Cre* mice (Sato et al., 2007). The Dkk3 promoter-driven expression of the Cre recombinase (Cre) takes place in retinal progenitors at E10.5, and is able to convert the *Top2b*^flox2^ allele to the *Top2b^−^* allele in retinal progenitors and their progenies. cKO (*Dkk3-Cre;Top2b*^flox2/flox2^) mice were viable and immunohistochemistry confirmed that no Top2b expression was detected in postnatal cKO retinas (supplementary material Fig. S2D). Initial examination showed no obvious morphological defects in the eyes between cKO mice and their control littermates before postnatal day 7 (P7); however, retinal degeneration accompanied with reduced eyeball size was observed starting from P14 (supplementary material Fig. S5). The openings of eyelids appeared to be narrower in cKO mice at P14 and P21 (data not shown); and closed completely in adult cKO mice (P180) (supplementary material Fig. S2F). Moreover, degeneration of the discontinuous circumferential folds in the collarette of iris of the cKO mice was observed, resulting in a smoother edge of the iris and larger pupils after P0 (supplementary material Fig. S5E–O). No visible iris structure can be found in adult cKO eyes (supplementary material Fig. S5S). In addition, optic nerves of the cKO mice were thinner and flatter (supplementary material Fig. S5D,H,L,P,T) as compared with the cylinder-like optic nerve of their control littermates. These results indicate that Top2b deficiency caused degeneration within the inner structure of the eyeballs; and prompted us to further examine the detailed structure and organization of the retina.

### Top2b deletion leads to defects in retinal lamination

**Fig. 2.**
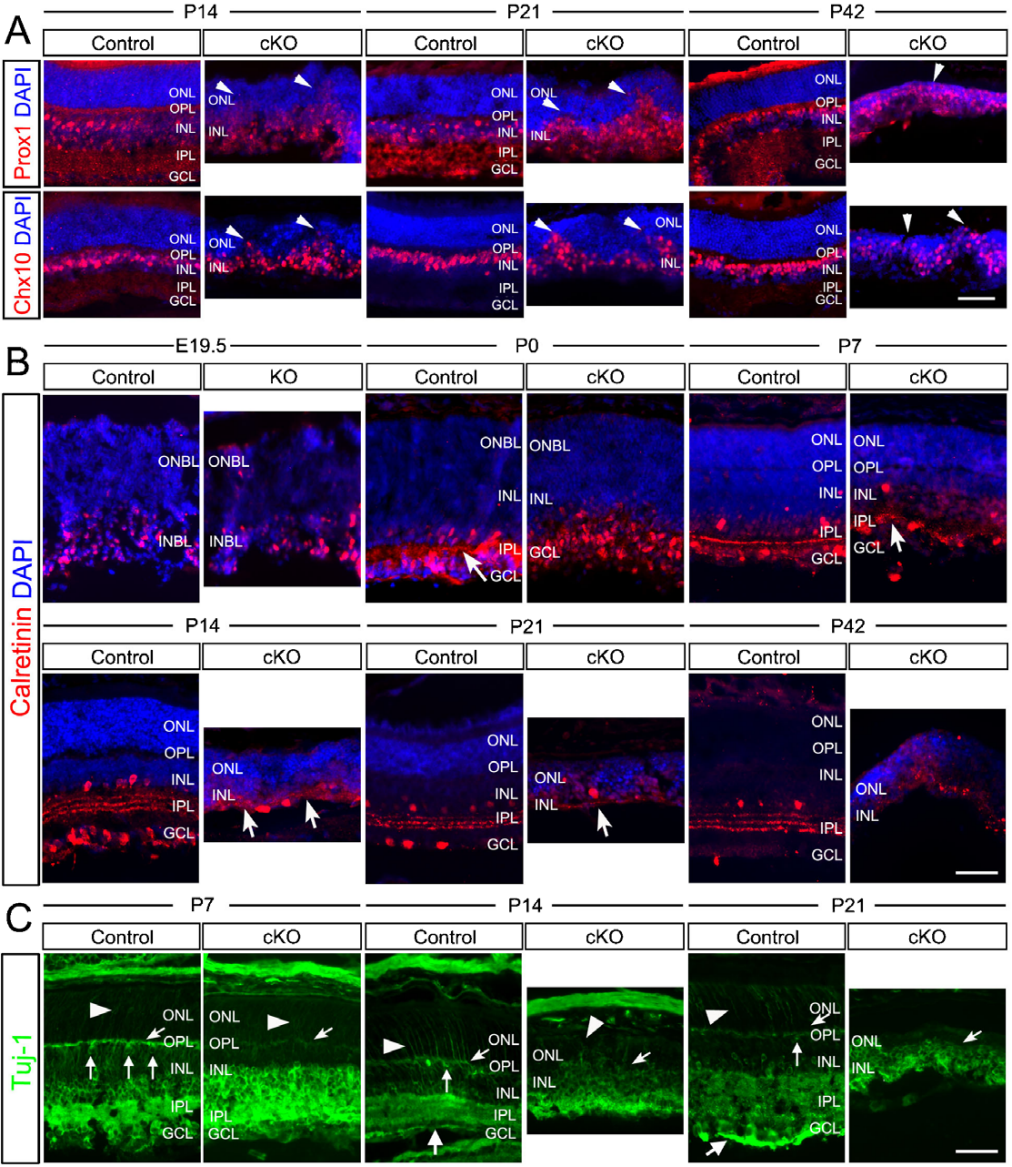
Aberrant retina lamination and loss of plexiform layers caused by Top2b deletion. (A) Retina sections prepared from P14, P21 and P42 control and Top2b cKO mice were stained with DAPI and co-stained with either Prox1 or Chx10. Prox1+ and Chx10+ cells were detected in the ONL (arrowheads) in Top2b cKO retinas. (B) Co-staining of E19.5-P42 control and KO/cKO retina sections with DAPI and Calretinin, which labels neurofilaments in the IPL. In cKO retinas, only a few filament-like structures were found at P7, P14 and P21, while in control samples the strata structure of neurofilaments appeared as early as P0 (arrows). (C) Co-staining with Tuj-1in P7, P14 and P21 control and cKO retina sections. Tuj-1 stained neurofilaments of the OPL (arrows) and processes of photoreceptors (arrowheads), which were largely missing in cKO samples. GCL, ganglion cell layer; INL, inner nuclear layer; IPL, inner plexiform layer; ONL, outer nuclear layer; OPL, outer plexiform layer. Scale bars: 50 µm.

To determine the cause of these morphological defects observed in the postnatal cKO mice, we next examined the cKO retinas at the cellular and molecular level. Retinal sections were stained with DAPI and various cell-specific markers, e.g. Prox1, Chx10, and Calretinin for retinal cells in the INL. Compared with controls, Prox1+ amacrine cells and Chx10+ bipolar in the cKO retinas did not retain in the INL, but protruded into the ONL (Fig. 2A). This could be explained by the largely missing inner plexiform layer (IPL) and the outer plexiform layer (OPL) in the cKO retinas as revealed by the Calretinin staining (Fig. 2B). Calretinin mainly stains the neurite plexuses of the IPL (formed by the processes of the amacrine cells, bipolar, and ganglion cells). In the controls, Calretinin-stained IPL was prominently present starting from P0 (Fig. 2B, arrow). The matured IPL contained five strata separating from each other by three plexuses could be observed at P14, P21 and P42 (Fig. 2B). However, in the cKO retinas, Calretinin staining revealed only short and disoriented neurites or processes and no plexus was visible at any postnatal stages examined (Fig. 2B). These defects became progressively more severe at P14, P21 and the adult stage (P42). The disappearance of the plexiform layers, especially the OPL, was further confirmed by the staining of the type-III β tubulin using the Tuj-1 antibody (Fig. 2C). In the control, both the OPL and IPL were clearly visible by P7; while in the cKO retinas, these plexiform layers were dramatically reduced by P7 and disappeared by P14 (Fig. 2C, arrows). These results suggest that Top2b is required for neurite outgrowth and proper formation of retinal plexiform layers.

### Top2b deficiency causes delayed differentiation of ganglion and horizontal cells, and affects their survival

**Fig. 3.**
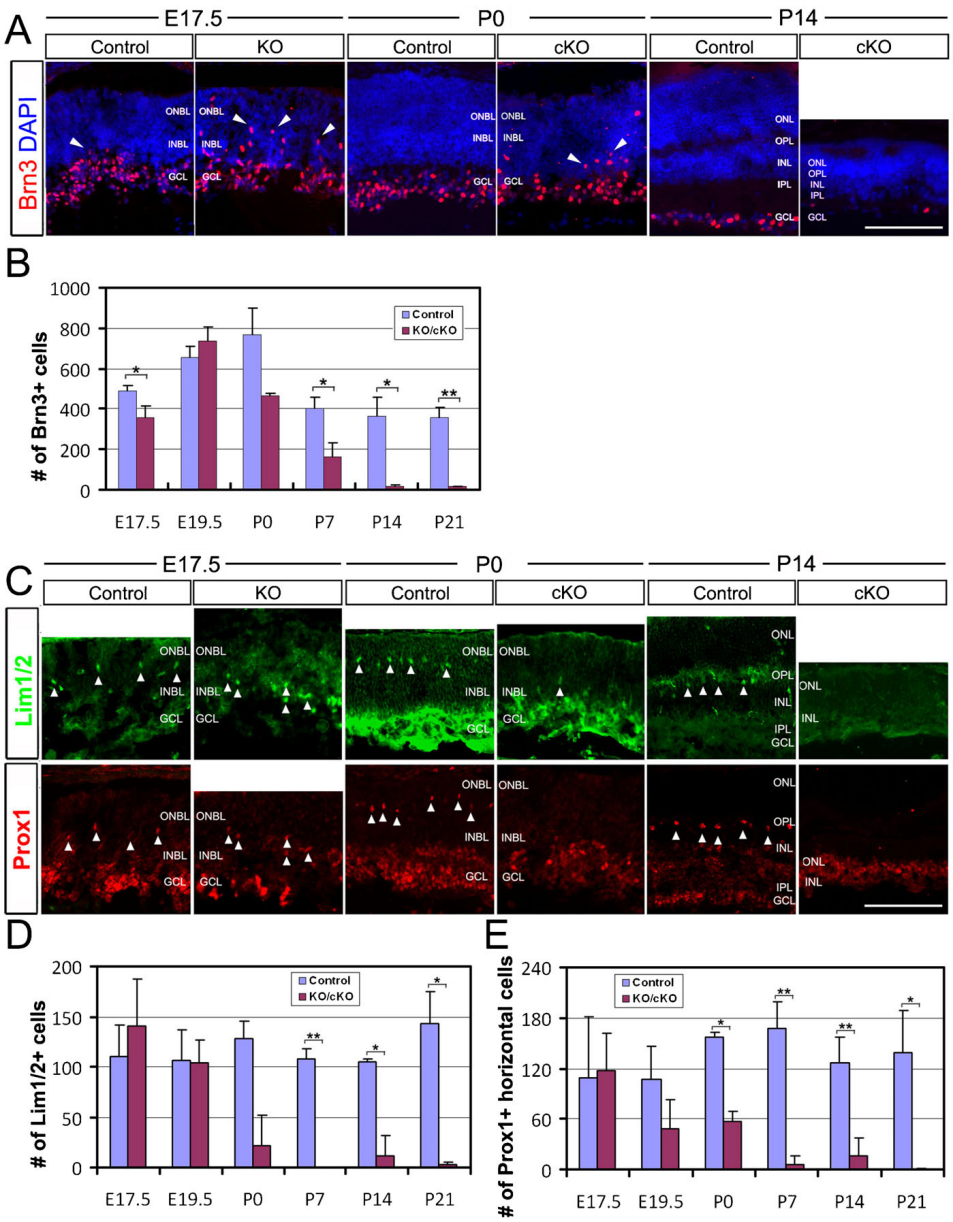
Top2b deficiency leads to delayed embryonic development and decreased ganglion and horizontal cells. (A) Retina sections of E17.5 control and KO embryos, and P0 and P14 control and cKO mice were co-stained with Brn3 and DAPI. Brn3+ cells were all located in the GCL in the control retinas, but they were more widely distributed in the INBL and ONBL (arrowheads) in the KO/cKO retinas. (B) Quantification of Brn3+ cells. A significant reduction in the number of Brn3+ cells in postnatal stages starting from P7 was observed. (C) Retina sections of E17.5 control and KO embryos and P0 and P14 control and cKO mice were co-stained with Lim1/2 and DAPI or Prox1 and DAPI. Lim1/2+ and Prox1+ horizontal cells were well-spaced and located in the horizontal cell layer (arrowheads) in the control retinas; while in the KO/cKO retinas, the majority of these cells remain in the INBL (arrowheads) at E17.5 and P0. By P14, no Lim1/2+ or Prox1+ cells were detected in cKO samples. (D) Quantification of Lim1/2+ cells. (E) Quantification of Prox1+ horizontal cells. Error bars are s.d. (*n* = 3 except *n* = 2 for P0). Student's t-test, *p<0.05, **p<0.01. INBL, inner neuroblastic layer; ONBL, outer neuroblastic layer; GCL, ganglion cell layer; INL, inner nuclear layer; IPL, inner plexiform layer; ONL, outer nuclear layer; OPL, outer plexiform layer. Scale bars: 100 µm.

Since the IPL is formed by the processes of INL cells (horizontal, amacrine and bipolar cells) and ganglion cells, defects in IPL could indicate that although Top2b deficiency does not affect INL and ganglion cell specification, but the final maturation and differentiation of these cell types was not accomplished. We thus further tracked the development of these retinal cells based on the expression of cell-specific differentiation markers with their laminar location and cell numbers. In KO/cKO retinas, the expression of Brn3 in ganglion cells, Lim1/2, Prox1 in horizontal and amacrine cells, and Chx10 in bipolar neurons was detected (Figs 2, 3), suggesting that specification of these retinal neuronal cell types was not affected in the absence of Top2b. However, Brn3+, Lim1/2+, and Prox1+ cells were widely dispersed in both the INBL and ONBL at E17.5 and P0 and appeared to be still undergoing the migration process in the INBL before reaching their final location in the horizontal cell layer (Fig. 3A,C). These results suggest that Top2b is involved in the process of terminal maturation/differentiation of these retinal cells.

In addition, there was a dramatic decrease in the number of Brn3+ ganglion cells in the cKO retinas during postnatal retinal development starting from P7, and by P14 only a few Brn3+ cells were detectable in cKO retina sections (Fig. 3B). This dramatic ganglion cell loss is consistent with the finding that cKO mice in late postnatal stages had thinner and flatter optic nerve (supplementary material Fig. S5), which is formed by axons emanated from ganglion cells. For Lim1/2+ and Prox1+ cells, a dramatic decrease in the number of these cells was observed in cKO retinas starting from P7 (Fig. 3D,E). These results suggest that Top2b deficiency affects terminal differentiation during embryonic retinogenesis, and retinal cell survival after birth.

### Absence of Top2b leads to defects in Müller glia development

**Fig. 4.**
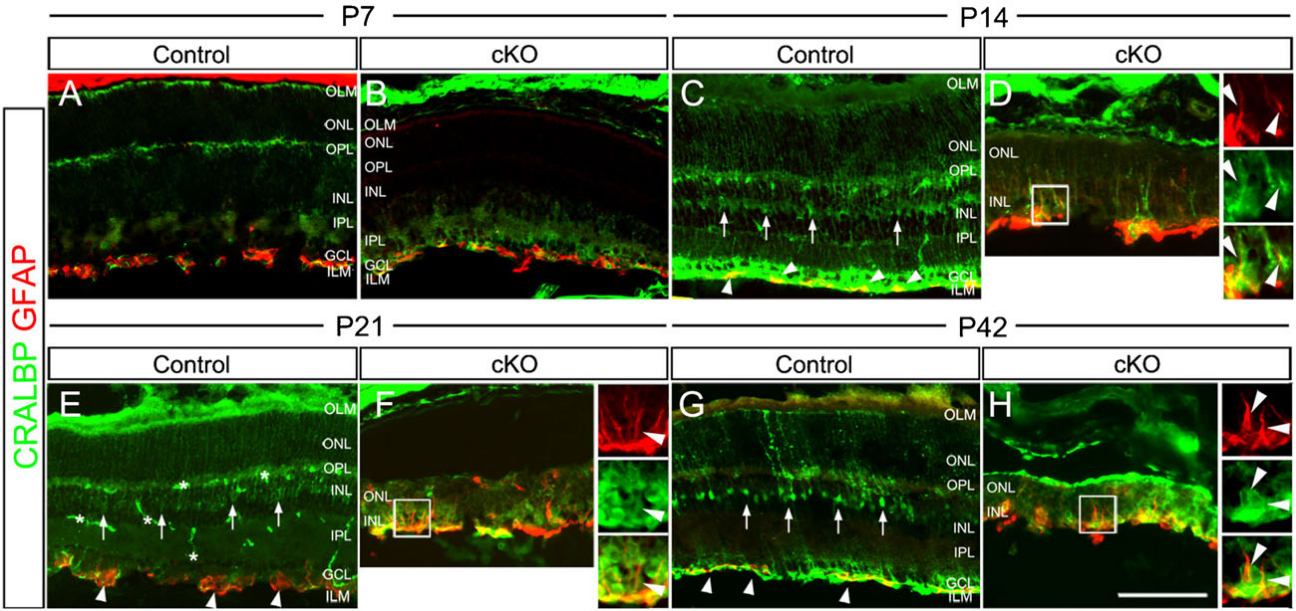
Top2b deficiency causes defects in Müller glia. Co-staining of control and cKO retina sections with GFAP and CRALBP at P7 (A,B), P14 (C,D), P21 (E,F) and P42 (G,H). At P7, GFAP expression (red) was present in the endfeet of Müller glia cells in both control and cKO retinas, while CRALBP-stained neurofilaments in the OPL were seen in the control retina (green in panel A) but not in cKO retina (B). Up-regulation of GFAP (arrowheads) accompanied with shorter processes and disrupted lamination was seen in cKO retinas at P14 (D), P21 (F) and P42 (H). CRALBP-stained processes and cell body of Müller glia (vertical arrows) were seen in the control retinas but not in the cKO at these stages (C–H). Asterisks indicate the background blood vessels. ILM, inner limiting membrane; OLM, outer limiting membrane; GCL, ganglion cell layer; INL, inner nuclear layer; IPL, inner plexiform layer; ONL, outer nuclear layer; OPL, outer plexiform layer. Scale bar: 100 µm.

Müller glia play important roles in the retina by supporting and nourishing other retinal cells (Poitry et al., 2000). Thus, there may be defects in Müller glia that contribute to the observed structural defects and cell loss in the cKO retinas. To examine the role of Top2b in Müller glial development, retinal sections were stained with radial glial marker GFAP and Müller glia marker CRALBP. In both the control and cKO retinas, GFAP signal was detected in the inner half of the retina at P7, where the endfeet of Müller glia cells are located (Fig. 4A,B). Starting from P14, CRALBP staining could be detected. Unlike the pattern found in control retinas in which CRALBP-stained bodies of Müller glia were observed to reside in the INL with full length processes expanding from the inner limiting membrane (ILM) to the outer limiting membrane (OLM) from P14 to P42 (Fig. 4C,E,G), there was no clear presence of Müller glia cell bodies in cKO retinas (Fig. 4D,F,H). In addition, CRALBP-stained processes of Müller glia in cKO retinas were shorter (not expanding to the OLM) and almost no processes were detected at P21 (Fig. 4D,F,H). GFAP staining in control retinas maintained in the endfeet of Müller glial throughout P14 to P42, while in cKO retinas it was stronger and more extensive (Fig. 4C–H). The GFAP staining in cKO retinas was found mostly close to the ILM and extended to co-localize with the processes of CRALBP+ Müller glia (Fig. 4D,F,H). Although this GFAP staining pattern is a characteristic feature of injury-induced reactive gliosis (Guérin et al., 1990; Fisher et al., 1995), the true nature of this observation requires further analysis. These results suggest that Top2b is essential for the development of Müller glia.

### Lack of Top2b affects the differentiation/maturation of photoreceptor cells

**Fig. 5.**
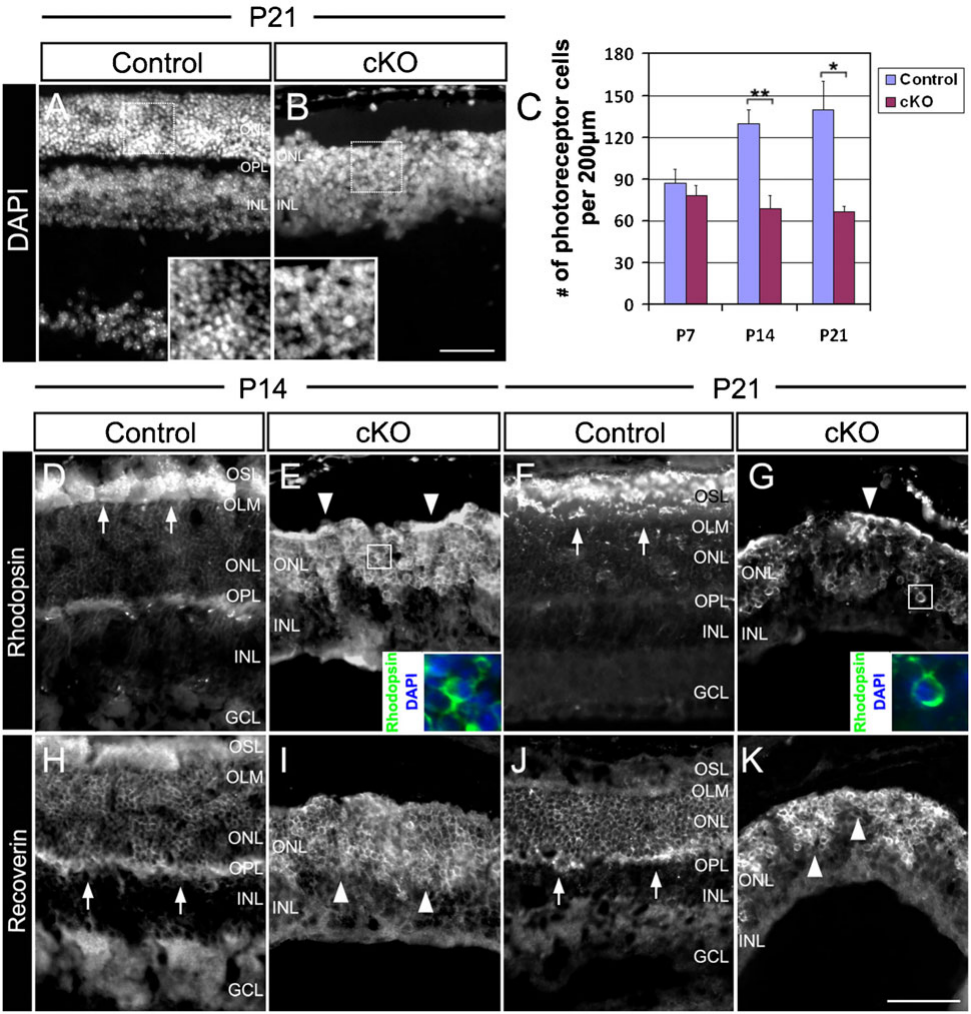
Top2b deletion leads to a decreased number of photoreceptors and loss of their outer segments. (A,B) P21 retina sections of control and cKO mice were stained with DAPI. The cKO retina appeared much thinner as compare to the width of control retina. The nuclei of cells in ONL of cKO retinas were also larger than those in control retinas (enlarged). (C) Quantification of the number of ONL cells (i.e. photoreceptors) showed a dramatic decrease of these cells in cKO retinas at P14 and P21. Error bars are s.d. (*n* = 3). Student's t-test: *, p<0.05; **, p<0.01. (D–G) Rhodopsin staining of P14 and P21 retina sections. Rhodopsin-stained outer segment layer (OSL) was detected in the control retinas (arrows), but not in cKO retinas (arrowheads). Instead, co-staining of Rhodopsin and DAPI (inserts in E and G show higher magnification of the boxed regions) of cKO retinas indicates the expression of Rhodopsin in the cytoplasm of ONL cells. (H–K) Recoverin staining of P14 and P21 retina sections. Recoverin stained outer plexiform layer (OPL, arrows) in the controls, but not in cKO retinas (arrowheads). GCL, ganglion cell layer; INL, inner nuclear layer; IPL, inner plexiform layer; ONL, outer nuclear layer; OPL, outer plexiform layer; OLM, outer limiting membrane; OSL, outer segment layer. Scale bars: 50 µm.

To further determine whether Top2b is required for photoreceptor development, retina sections were stained with photoreceptor cell markers Rhodopsin (rod outer segment) and Recoverin (rod, cone and cone-bipolar cells). Rhodopsin+ and Recoverin+ cells were detected in both the control and cKO retina (Fig. 5), indicating that the absence of Top2b did not affect photoreceptor cell fate specification. In cKO retinas, however, the DAPI-stained nuclei in the ONL appeared to be larger and more loosely distributed (Fig. 5A,B), and the number of ONL cells were significantly reduced starting from P14 (Fig. 5C). In addition, rhodopsin staining revealed irregularly clustered cell bodies in the ONL, and there was no discernible outer segment layer (OSL) in the cKO retinas (Fig. 5E,G). Instead of being expressed in outer segments, Rhodopsin was found in the cytoplasm of these cells in ONL of cKO retinas (Fig. 5E,G, enlarged). These phenotypes are indicative of photoreceptor degeneration (Fariss et al., 1997; Louie et al., 2010). In addition, Recoverin staining revealed that both the OSL and plexiform layers were largely missing in the cKO retinas (Fig. 5I,K). These results suggest that Top2b is required for the development of the OS and maintenance of photoreceptors.

### Top2b deletion increases retinal cell death

**Fig. 6.**
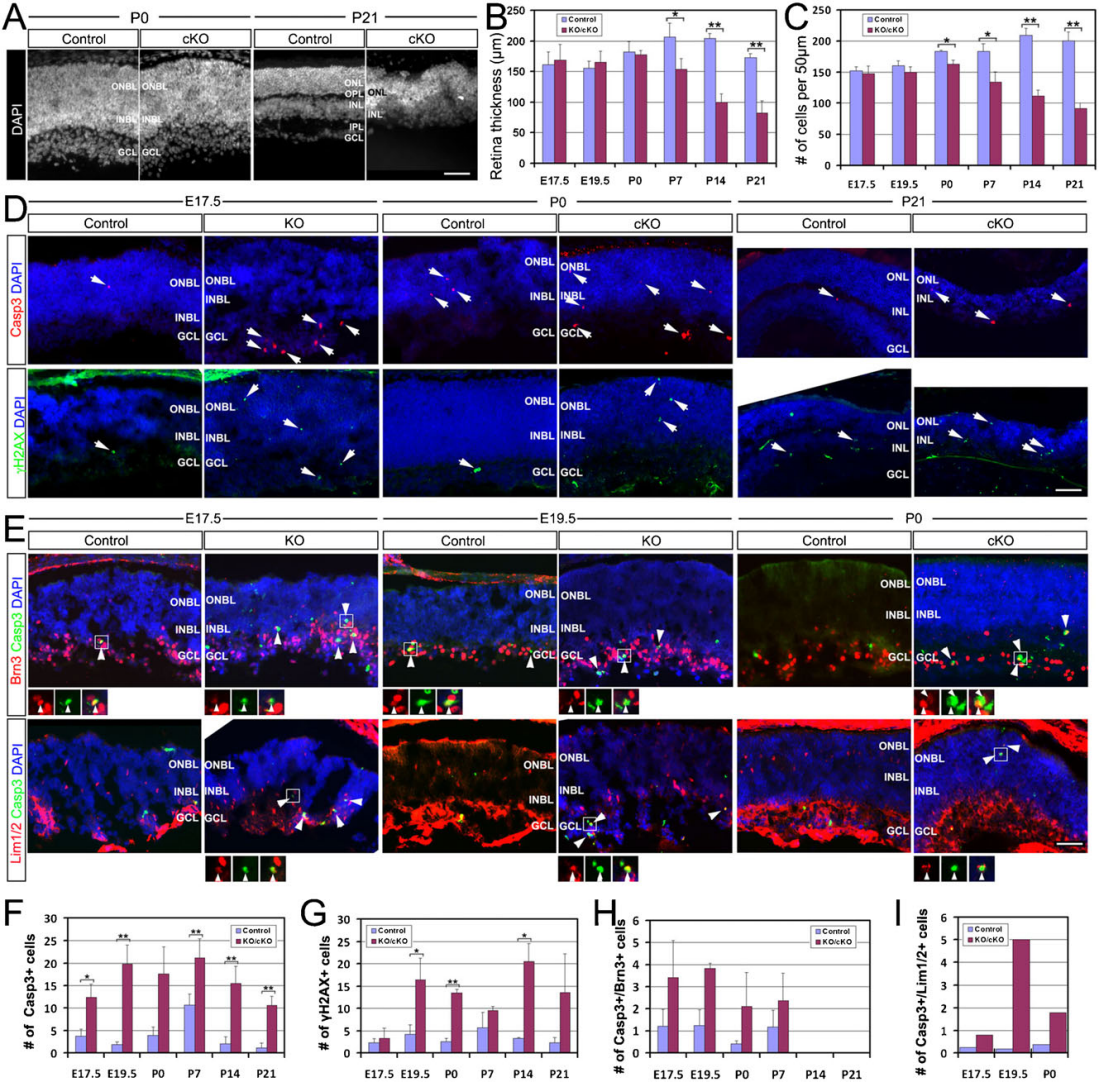
Increased cell death in Top2b deficient retinas. (A) DAPI stained retinal nuclei at P0 and P21. Quantification showed a significant decrease in both retina thickness (B) and the average number of cells (C) in cKO retinas starting from P7 and P0, respectively. (D) Co-staining of retina sections with cleaved caspase 3 (Casp3) and DAPI or γ-H2AX and DAPI. There was a significant increase in the number of Casp3+ and γ-H2AX+ cells in Top2b deficient retinas (D, arrows, F,G). (E) Co-staining of retina sections with Casp3 and Brn3, or Casp3 and Lim1/2. There was a significant increase in Casp3+/Brn3+ and Casp3+/Lim1/2+ cells (E, arrowheads, H,I) in KO/cKO retinas. Error bars are s.d. (*n* = 3); *p<0.05; **p<0.01. INBL, inner neuroblastic layer; ONBL, outer neuroblastic layer; GCL, ganglion cell layer; INL, inner nuclear layer; ONL, outer nuclear layer. Scale bars: 50 µm.

Examination of DAPI-stained cKO retina sections showed that there was a significant reduction in retina thickness starting from P7 (Fig. 6A,B), in retinal perimeter starting from P14 (supplementary material Fig. S6), and in cell number starting from P0 (Fig. 6A,C). This could be due to either a decrease in cell proliferation or an increase in cell death, or both. Since Top2b expression was absent from proliferating retinal progenitors (Fig. 1A), it is unlikely that Top2b deficiency affects progenitor proliferation. Indeed, we have confirmed that Top2b deficiency did not affect cell proliferation by S-phase labeling with EdU and M-phase labeling with PH3 (supplementary material Fig. S4A). There were no significant differences in the number and laminar locations of EdU+ or PH3+ cells between the control and KO retinas (supplementary material Fig. S4B,C); it suggests that Top2b deficiency does not affect retinal progenitor proliferation.

We next examined whether there was a difference in the cleavage orientation during cell division as it has been shown that cleavage orientation affects the cell fate decision of the daughter cells (Chenn and McConnell, 1995; Godinho et al., 2007; Arai et al., 2011), which affects the number of cells leaving cell circle. However, our analysis showed that there were no significant differences in the mitotic cleavage orientations of PH3/EdU double-labeled cells lining the apical surface of the retina (supplementary material Fig. S4E) between KO and control littermates. These findings, together with the absence of Top2b expression in proliferating (EdU+) cells, suggest that Top2b deficiency does not affect cell proliferation/division during retinogenesis.

Next, we examined whether Top2b deficiency causes increased retinal cell death by staining retina sections with apoptosis and DNA damage markers, cleaved caspase 3 (Casp3) and γ-H2AX, respectively. Cleavage of Casp3 is indicative of the ongoing programmed cell death known as apoptosis (Kuribayashi et al., 2006) and γ-H2AX is a surrogate marker for DNA double-strand breaks (Kinner et al., 2008; Kuo and Yang, 2008). The results showed that there was a significant increase in the number (2 to 6-fold) of Casp3+ and γ-H2Ax+ cells in KO/cKO retinas (Fig. 6D,F,G). Double immunostaining with Casp3 and retinal cell-specific marker Brn3 or Lim1/2 showed significantly increased cell death in the ganglion and horizontal cell population from E17.5 to P21(Fig. 6E,H,I). The retinas from P42 and P180 cKO mice degenerated so severely that we were unable to find an intact section for further analysis. Together, these results suggest that Top2b deficiency causes increased retinal cell death.

### Top2b deletion impairs transcription of genes associated with cell survival and neurological system development

**Fig. 7.**
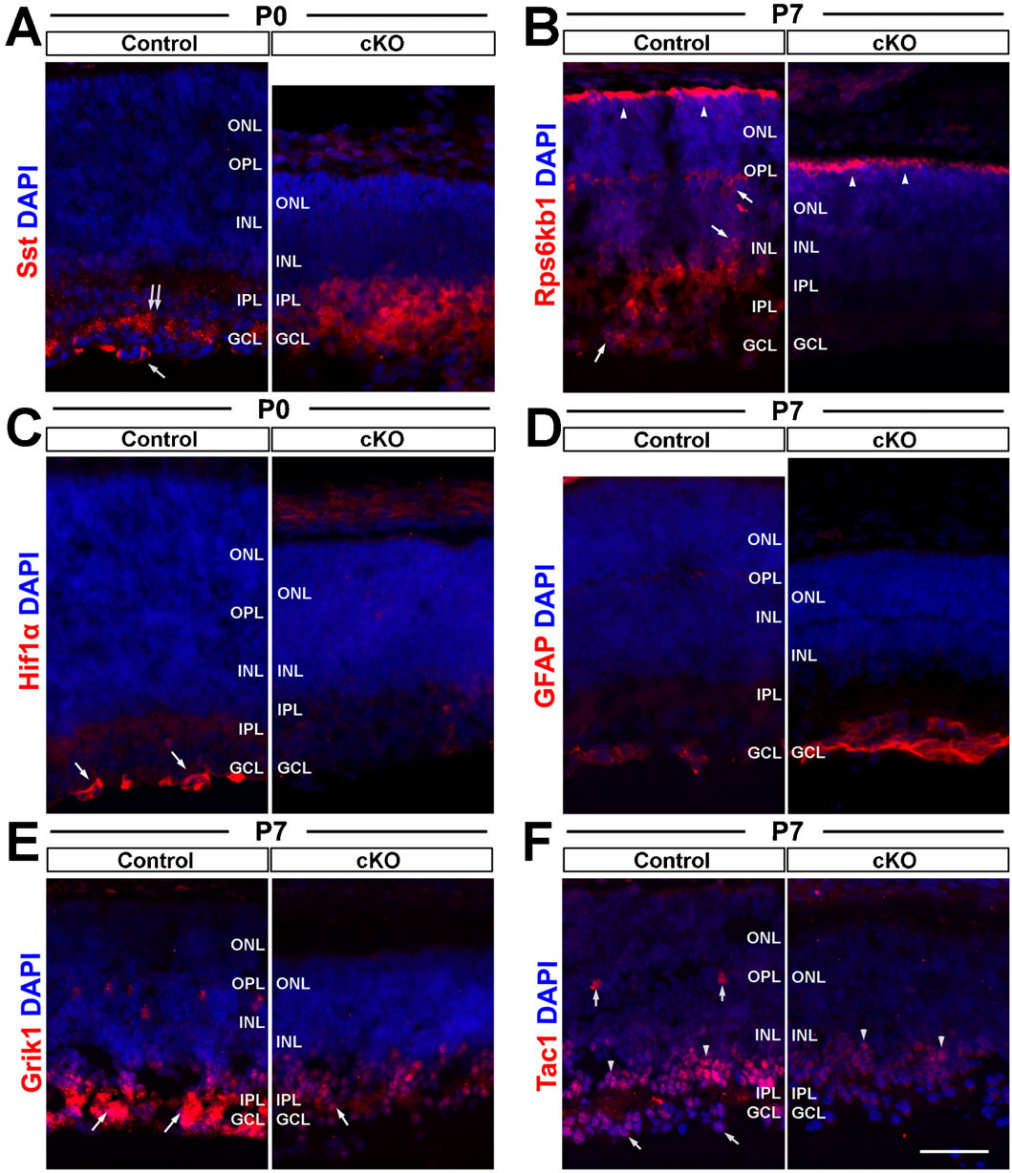
Top2b deletion affects the expression of genes involved in neural cell survival and neurite growth. Control and cKO retina sections of P0 and P7 mice were stained with antibodies against six proteins with their respective mRNA differentially expressed in cKO retinas. (A) Sst staining in cKO sample was increased but lost the specific immunoactivity in the GCL (double arrow) and ganglion cells (arrow) in the control retinas. (B) The Rps6kb1 protein was expressed in the outer segments of photoreceptors (arrowheads), ganglion cells, amacrine cells (arrows) and the IPL in the control retinas. However, its expression was only seen in the outer segments (arrowheads) in cKO retinas. (C) Hif1α was found in the cytoplasm of marginal ganglion cells in the controls (arrows), but was not detectable in the cKO samples. (D) Increased GFAP expression was found in GCL of P7 cKO retinas. (E) Grik1 was strongly stained in the IPL of the control retina, but reduced in the cKO retina (arrows). In addition, Grik1 staining in horizontal cells was missing. (F) Tac1 was found in horizontal cells (vertical arrows), amacrine cells (arrowheads) and ganglion cells (diagonal arrows) in the control retina. In the cKO retina, the signal was only found in amacrine cells with a decreased intensity. GCL, ganglion cell layer; INL, inner nuclear layer; IPL, inner plexiform layer; ONL, outer nuclear layer; OPL, outer plexiform layer. Scale bar: 50 µm.

To identify genes and gene networks that are responsible for the phenotypes observed in the postnatal Top2b cKO retinas, we employed the next generation RNA sequencing (RNA-seq) analysis. Total RNA was isolated from the retinas (pool of four retinas for each sample and time point) of control and Top2b cKO mice at P0 and P6, the two critical postnatal developmental stages when major defects in cKO retinas start to appear. After sequencing, ∼120 million 50 bp reads for each time point and each group were obtained and mapped to the mouse genome (MGI, as of Feb 15, 2012). Over 62,000 transcripts were identified; these include ∼22,000 annotated genes (including alternative-spliced variants), which cover 76% of the whole mouse genome. Among all the annotated genes, 8.80% (1,935/22,000) for P0 and 1.25% (274/22,000) for P6 showed differential expression between the control and cKO samples (p≤0.05, q-value ≤0.05). Further bioinformatic analysis revealed that these differentially expressed genes (DEGs) were associated with apoptosis, system development, cellular transportation, signal transduction at P0; and neurological system process and cytoplasm condition at P6 (supplementary material Table S1). Examples include Igf1, Hras, Mapk1, HIF1α, Vegfa, Rps6bk1, Sst, Tac1, Gfap, GriK1/2, Gria4, Grm7 and Nrxn1/3, which are involved in the mouse retinal development. The differential expression of some of these genes was confirmed by immunohistochemistry analysis (Fig. 7; supplementary material Table S1).

**Fig. 8.**
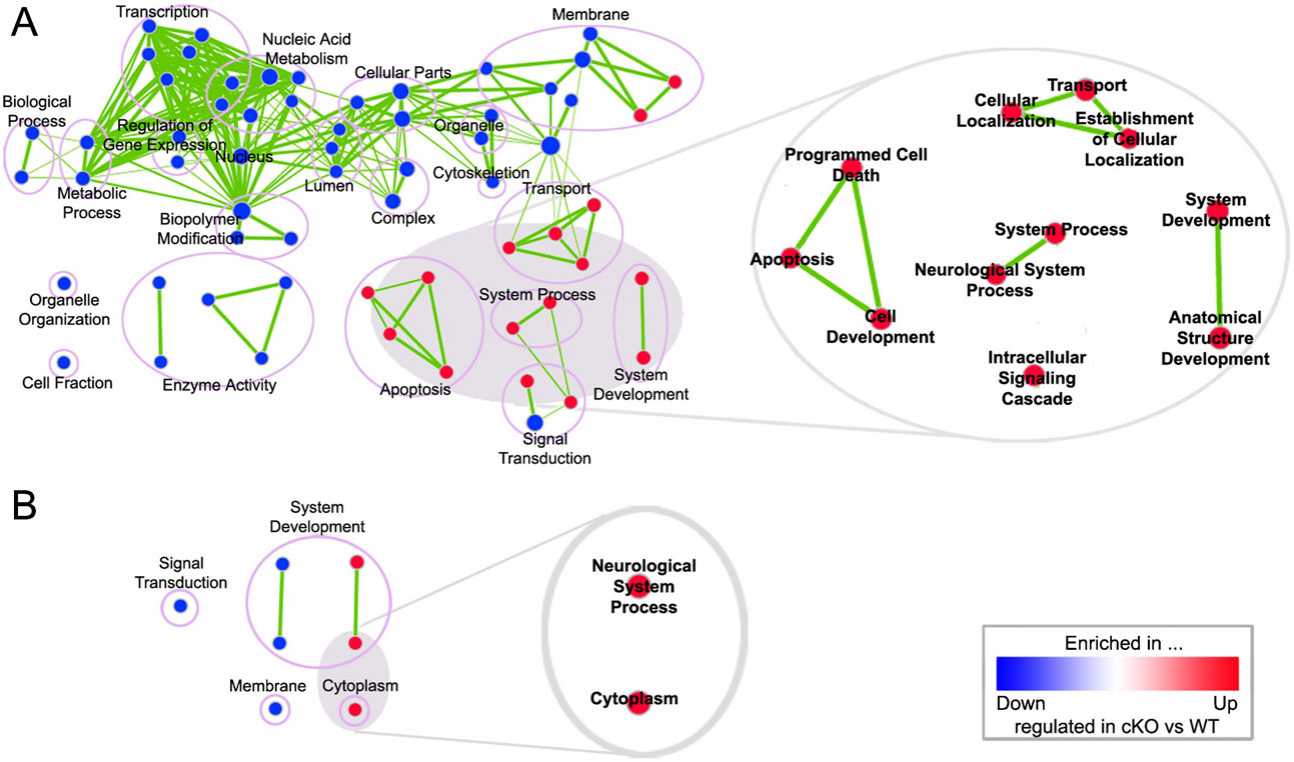
Gene ontology enrichment analysis reveals Top2b function in neurite growth and maintenance/survival of retinal cells. Differentially expressed genes were analyzed for gene ontology (GO) enrichment using GSEA. The result was mapped on a network of gene-sets (nodes) connected by their similarity (edges) for P0 (A) and P6 (B) using Cytoscape (p≤0.005, FDR q-value ≤1, and similarity ≤0.5). Nodes highly enriched with up-regulated genes in cKO samples are shown in red while those with down-regulated genes are shown in blue. Node size represents the gene-set size. Edge thickness represents the degree of overlap between two gene-sets. Nodes were grouped according to GO definition and annotated with the group names. The cluster of red nodes under restricted cut-off values (p≤0.005, FDR q-value ≤0.1, and similarity ≤0.5) was enlarged, showing that cell death, cellular transportation and neurological system development related processes were highly enriched in cKO samples.

Further functional enrichment analysis using GSEA (Gene Set Enrichment Analysis, v2.07) (Subramanian et al., 2005) revealed that these DEGs were enriched in processes related to neural system development, apoptosis, transportation, and signal transduction (Fig. 8; supplementary material Table S2), confirming an important role of Top2b in retinal cell survival and maintenance.

## Discussion

In this study, using the traditional constitutive Top2b KO and retina-specific Top2b cKO mouse models and in combination with the RNA-seq analysis, we report the functional role of Top2b in mammalian retina. We demonstrated that Top2b is required for terminal differentiation and proper laminar structure formation but not for cell fate specification during embryonic retinal development. In addition, we have further revealed the important function of Top2b in cell survival/maintenance in the postnatal retina. Top2b is expressed in all differentiating and mature retinal cell types; and as a DNA enzyme known to involve in transcription regulation, retinogenesis phenotypes observed in Top2b-deficient retinas are likely the consequence of altered gene expression. Our genome-wide transcriptome analysis confirmed that the differentially expressed genes in Top2b-deficient retina are associated with neurological system development and cell survival.

Previous studies including our own have shown that Top2b deficiency does not affect cell proliferation in cultured mouse embryonic fibroblasts (Lyu and Wang, 2003; Lin et al., 2013) and mouse embryonic stem cells (Tiwari et al., 2012). Consistent with these observations, our *in vivo* data obtained from Top2b KO retinas showed no difference in the S-phase labeling with EdU, M-phase labeling with PH3, or mitotic cell cleavage orientations (supplementary material Fig. S4). Together with the mutually exclusive pattern of EdU labeling and Top2b expression (Fig. 1A), this study confirms that Top2b is unlikely to be involved in cell proliferation *in vivo*.

Although there were severe lamination defects and neurodegeneration in Top2b-deficient retina, all retinal cell types (i.e. ganglion, horizontal, amacrine, bipolar, rod photoreceptor, cone photoreceptor, and Müller glial cells) have emerged (Fig. 1B–H; supplementary material Fig. S3). The timing of the expression of cell-specific makers seems to follow a normal generation timeline of the corresponding retinal cell types (Fig. 1I). These findings demonstrate that Top2b does not affect cell cycle withdrawal and cell fate determination in retinal progenitors *in vivo*; and are consistent with the notion that cell fate specification is determined during the last cell cycle of neuronal progenitors before their asymmetric cell division (McConnell, 1991; McConnell and Kaznowski, 1991).

In our previous studies, the lack of Top2b was found to cause aberrant lamination in the mouse cerebral cortex, which may be resulted from the decreased Reelin expression (Lyu and Wang, 2003). Interestingly, we found that Top2b deficiency in the retina causes a delayed differentiation phenotype in ganglion cells, horizontal cells, a lack of OS in photoreceptors, and severe defects in the plexiform layers, indicating a role of Top2b in cell differentiation, neurite outgrowth and proper retinal laminar formation. RNA-seq analysis revealed a decreased expression level of several migration guidance molecules in the cKO retinas, including Reelin. Since Reelin plays important role in guiding cell migration and positioning in laminar structures (Rice and Curran, 2001), the reduction of its expression could explain the mis-positioned horizontal and ganglion cells (Fig. 3). However, although Reelin KO mice (Reeler mice) showed a disruption in synaptic circuitry formation in the IPL and decreased rod bipolar cell density, the IPL structure can still be identified even in the adult animals (Rice et al., 2001). Thus, the reduced Reelin expression itself is insufficient to cause the severe degeneration of the plexiform layers observed in Top2b cKO retinas.

Top2b deficiency not only tampers the target finding of axons (Yang et al., 2000) but also inhibits neurite growth in two levels: shorter neurite lengths and growth cone degeneration (Nur-E-Kamal et al., 2007). In addition, Top2b has been identified as the main regulator of ganglion cell axon path finding during zebrafish retinal development by a forward genetic screen (Nevin et al., 2011). Although the molecular mechanism underlying the role of Top2b in neurite growth remains to be determined, it is clear that the disrupted neurite growth caused by Top2b deletion can contribute to the aberrant lamination as well as loss of synaptic connections in the IPL, OPL, and the degeneration of neurofilaments in Top2b cKO retina (Figs 2, 5).

We showed that during postnatal retinal development, Top2b is necessary for the survival of postmitotic retinal cells, including ganglion, horizontal, photoreceptor cells, Müller glia, and possibly other cell types. Lack of Top2b results in a significant increase in the number of cells that contain DNA damage and/or apoptotic signals (γ-H2AX+ and Casp3+) (Fig. 6), smaller eyeballs, and the thinner optic nerves (supplementary material Figs S5, S6). The findings presented here are coherent with a critical role of Top2b in neuronal survival and maintenance *in vivo*, and with the previous report that Top2b is required for the survival of mouse embryonic stem cell-derived neurons in culture (Tiwari et al., 2012). The interpretation that Top2b plays a critical role in postmitotic retinal cells survival/maintenance may well be extrapolated to neurons located in other parts of the CNS. Interestingly, it has been reported that Top2b level decreases as neurons age (Bhanu et al., 2010) and Top2b deficiency can lead to dopaminergic neuron degeneration (Heng et al., 2012), further implicating a plausible role of Top2b in CNS degenerative diseases.

Consistent with the observed phenotypes, our RNA-seq analysis revealed that the differentially expressed genes (DEGs) in Top2b cKO retinas are associated with cell survival and neurological system development (Fig. 8; supplementary material Tables S1, S2). For instance, Igf1 is known to be a key regulator that promotes growth and development, and can induce the differentiation of ganglion cells, rod photoreceptors and one subtype of glial cells (Meyer-Franke et al., 1995; Fischer et al., 2010; Pinzon-Guzman et al., 2011). The down-regulation of Igf1 pathway and other pathways such as Erk (Hras and Mapk1), Hif1a, Vegf, and Atk (Rps6bk1) were known to affect cell survival (Dudek et al., 1997; Jin et al., 2002; Chang et al., 2003; Tomita et al., 2003), which may explain the dramatic cell loss in the cKO retinas. Other DEGs such as Tac1, GriK1/2, Gria4, Grm7 and Nrxn1/3 are important neurotransmitters and receptors (Dingledine et al., 1999; Millán et al., 2002; Bagnoli et al., 2003; Uemura et al., 2010), which are involved in modulating myriad aspects of neuronal function. The up-regulation of Sst at P0 and down-regulation of Tac1 at P6 in cKO retinas can impact neuronal function and survival due to their essential role as neurotransmitters in the retinal circuits (Bagnoli et al., 2003). Sst inhibits cell proliferation (Lahlou et al., 2003) but aid neurite growth (Kungel et al., 1997). Tac1 prevents neuronal damage during development and enhances nerve growth factor-mediated neurite outgrowth (Bagnoli et al., 2003). Thus, the up-regulated Sst and down-regulated Tac1 could alter the state of retinal development in the same direction. Moreover, both factors are localized to amacrine and ganglion cells (Zalutsky and Miller, 1990; Cristiani et al., 2002; Catalani et al., 2006) where major defects were found in Top2b KO/cKO retinas.

The question of how Top2b deficiency causes differential expression of particular genes remains largely unanswered. Although we cannot rule out the possibility that the increased retinal cell death may contribute to the differential expression of specific genes, however, a series of *in vivo* and *in vitro* studies have supported the claim that Top2b is a crucial player in regulating transcription (Tsutsui et al., 2001a; Lyu and Wang, 2003; Ju et al., 2006; Lyu et al., 2006; Tiwari et al., 2012; King et al., 2013). Top2 can modulate DNA topology and chromatin structure by performing DNA strand passage reactions. The Top2a isozyme, which is only expressed in proliferating cells, is known to orchestrate sister chromatin segregation and chromatin condensation/decondensation in association with other chromatin-modulating enzymes such as condensin (Lupo et al., 2001). The absence of Top2a and presence of Top2b in postmitotic cells undergoing terminal differentiation may imply that Top2b can regulate gene expression by controlling local chromatin structure. Recently, the possible role of Top2b in regulating transcription near the promoter has gained further support from studies of identifying unconstrained DNA supercoiling as well as promoter melting during transcription activation (Kouzine et al., 2013a; Kouzine et al., 2013b; Naughton et al., 2013). However, the molecular mechanism that underlies Top2b function in this respect still requires further investigation.

In summary, our studies have demonstrated that Top2b plays an essential role in the survival and maintenance of postmitotic retinal cells. In the absence of Top2b, proper retinal development is affected starting at early postnatal stages. Top2b-dependent genes identified by RNA-seq are known to control neuronal survival and neurite outgrowth. Identification of genes that are directly controlled by Top2b will be the next step in unveiling the molecular mechanism of Top2b in transcription regulation.

## Materials and Methods

### Mouse strains

The traditional constitutive Top2b knockout (KO) mice were described in a previous publication (Lyu and Wang, 2003). The retina-specific Top2b knockout (cKO) mice were generated by crossing the *Top2*b^flox2^ mouse strain (Lyu and Wang, 2003) with the *Dkk3-Cre* mouse strain (Sato et al., 2007) (kindly provided by Dr Hiromi Sesaki of the Johns Hopkins University). *Dkk3-Cre* mice showed restricted Cre expression to retinal progenitors starting from E10.5 (Sato et al., 2007). Based on our specific mating scheme (*Top2b*^flox2/flox2^ × *Dkk3-Cre;Top2b*^+/flox2^), the control mice used in this study had genotypes of *Dkk3-cre;Top2*b^+/flox2^, *Top2*b^+/flox2^, or *Top2*b^flox2/flox2^ and the retina-specific Top2β knockout mice had the genotype of *DKK3-Cre*;*Top2*b^flox2/flox2^. The *Dkk3-Cre*;*Top2*b^flox2/flox2^ (cKO) mice were genetically *Top2*b^−/−^ in retinal progenitors and all cells derived from them. PCR-based genotyping of the *Top2b*^+^ (primers PR3 and PR1) and *Top2b*^flox2^ (primers PR3 and PR4, PR5 and PR6) alleles was performed using mouse tail DNAs as described previously (Lyu and Wang, 2003) and the *Cre* allele was amplified using primers Cre3 (5′-CACCCTGTTACGTATAGCCG-3′) and Cre4 (5′-GAGTCATCCTTAGCGCCGTA-3′).

### Tissue preparation

Eyes of control or Top2b KO/cKO mice were dissected immediately after mice were sacrificed. They were washed in 1× PBS and fixed with 4% (w/v) paraformaldehyde for 1 hr. Fixed tissues were washed again and then cryopreserved in 30% (w/v) sucrose overnight. Images of the whole-mount eyes were taken with a microscope (Leica, MZ16FA) and eyeball diameter was measured. The eyeballs were embedded in cryo-preserving medium (Tissue Tek® OCT compound) and kept frozen at −80°C.

### Immunohistochemistry

Frozen retinal tissues were sectioned sagittally (10–12 µm in thickness) using a cryostat (ThermoScientific) and air dried. Sections were blocked and permeablilized for 1 hr in blocking buffer containing 10% donkey serum, 0.1% Triton X-100, and 0.1% Tween® 20 at room temperature. Afterwards, they were incubated with primary antibodies overnight at 4°C. Following three 10-min washes in PBS, sections were incubated in the blocking buffer containing corresponding fluorophore-conjugated secondary antibodies for 1 hr at room temperature. Slides were then washed three times with PBS (10-min each wash), and mounted with mounting medium (Vector Laboratories) in the presence or absence of DAPI (to label the nuclei). The following primary antibodies were used: Top2b (1:300, sc-25330), Brn3 (1:300, sc-6026), Chx10 (1:300, sc-21692), Pkcα (1:300, sc-8393), EAAT1 (1:200, sc-7757), CRALBP (1:200, sc-59487) and PH3 (1:300, sc-8656-R) from Santa Cruz Biotechnology, Inc.; Lim1/2 (1:25) and Pax6 (1:15) from Developmental Studies Hybridoma Bank (DSHB); Brn3a (1:100, MAB1585), Calretinin (1:2500, MAB1568), Recoverin (1:1000, AB5585) and γ-H2AX (1:100, 05-636) from Millipore; Tuj-1 (1:1000, ab14545) from Abcam; cleaved-Casp3 (1:1600, no. 9661) from Cell Signaling; Prox1 (1:5000, prb-238c) from Covance; Rhodopsin 4D2 (1:100; gift from Dr Robert S. Molday from University of British Columbia, Canada). Images were captured using a Zeiss Axio Imager M1 fluorescence microscope and AxioVision 4.8.

### *In vivo* EdU labeling

To label proliferating cells *in vivo*, the thymidine analog EdU (5-Ethynyl-2′-deoxyuridine, 50 µg/g body weight, in PBS) was injected into pregnant female mice 2 hr before sacrifice and dissection. EdU labeling was detected using the Click-iT Edu Alexa Fluor 647 Imaging kit (Invitrogen).

### Whole genome transcriptome analysis using the RNA-seq method

Total RNA were isolated from retinas of the control and cKO pups at P0 and P6 (*n* = 4 for each sample). The whole transcriptome RNA libraries were constructed, following by deep sequencing using the SOLiD System (Applied Biosystems). RNA-seq data analysis was performed according to the published protocol (Trapnell et al., 2012) with minor modifications. Briefly, raw 50 bp reads was aligned to the mouse genome (MGI, as of Feb 15, 2012) using Bowtie (Langmead et al., 2009). Gene expression level was analyzed with Cufflinks, with differentially expressed genes determined by Cuffdiff (Trapnell et al., 2010). Gene Ontology (GO) analysis of the gene expression data was performed using GSEA (permutation  =  1000) (Subramanian et al., 2005) and visualized by the Enrichment Map tool (The Bader lab, University of Toronto) as a plug-in of Cytoscape (Merico et al., 2010).

### Cell counting

Cell counting was performed manually on retina sections through the central regions at or directly adjacent to the optic nerve level. Either the whole section or two 200 µm wide regions from the central part of the retina on each side of the optic nerve were counted, as indicated in the histograms.

### Statistical analysis

Quantitative data were presented as mean ± standard deviation. Significance (p-value) was determined by Student's t-test.

## Supplementary Material

Supplementary Material
