# Supplementary material for: Topoisomerase IIbeta is required for proper retinal development and survival of postmitotic cells
Source: Biol Open. 2014 Jan 17;3(2):172–84. doi: 10.1242/bio.20146767 (PMC3925320; doi:10.1242/bio.20146767)
Supplement: Supplementary Material [file supp_3_2_172__index.html]

Topoisomerase IIbeta is required for proper retinal development and survival of postmitotic cells — Topoisomerase IIbeta is required for proper retinal development and survival of postmitotic cells — Supplementary Material 

# Topoisomerase IIbeta is required for proper retinal development and survival of postmitotic cells

## bio.20146767 Supplementary Material

**Files in this Data Supplement:**

- Supplementary Material - Ying Li et al. doi: 10.1242/bio.20146767
