# Supplementary material for: Topoisomerase IIbeta is required for proper retinal development and survival of postmitotic cells
Source: Biol Open. 2014 Jan 17;3(2):172–84. doi: 10.1242/bio.20146767 (PMC3925320; doi:10.1242/bio.20146767)
Supplement: Supplementary Material [file supp_bio.20146767_bio.20146767-s1.pdf]

Supplementary Material

Ying Li et al. doi: 10.1242/bio.20146767

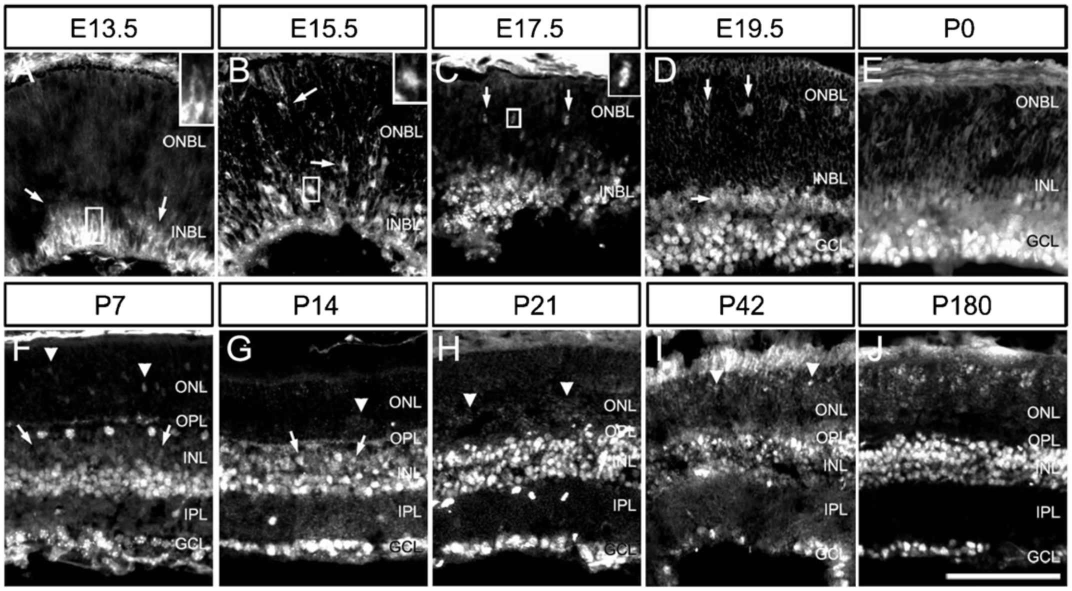

Fig. S1. Top2b expression during mouse retinal development. Retina sections from different developmental stages were stained with the anti-Top2b antibody. Top2b expression was first detected at E13.5 in the lower portion of the INBL (A) in the cytoplasm, and then extended to the upper INBL and lower ONBL at E15.5 (B, arrows) in the nuclei. Starting from E17.5, it appeared in the horizontal cells (C,D, vertical arrows), the cells in the INL and GCL (D–J, arrows), and photoreceptor cells (F–J, arrowheads). Top2b expression is maintained in the adult retina at P180 (J). Cellular identities of Top2b+ cells were determined by their position and double staining with cell-specific markers (Fig. 1). Boxed region is shown in a higher magnification. INBL, inner neuroblastic layer; ONBL, outer neuroblastic later; GCL, ganglion cell layer; INL, inner nuclear layer; IPL, inner plexiform layer; ONL, outer nuclear layer; OPL, outer plexiform layer. Scale bar: 100 µm.

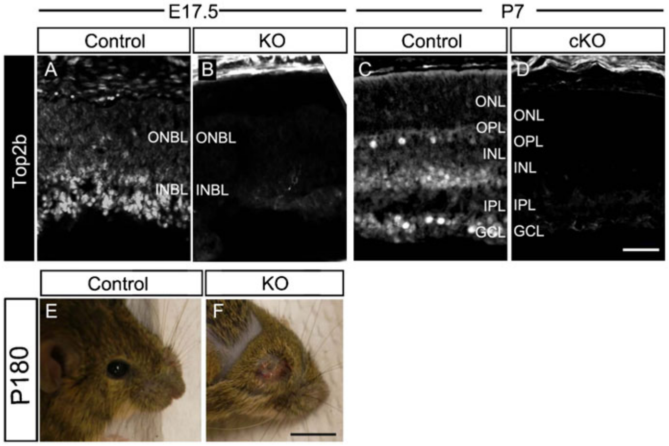

Fig. S2. Eye degeneration in Top2b deficient mice. (A–D) Top2b expression was detected in retinas of control littermates, but not in retinas of Top2b-deficient E17.5 KO or P7 cKO littermates. (E,F) Severe eye degeneration was observed in adult retina-specific Top2b KO (cKO) mice. Scale bars: 50 µm (A–D); 1 cm (E,F).

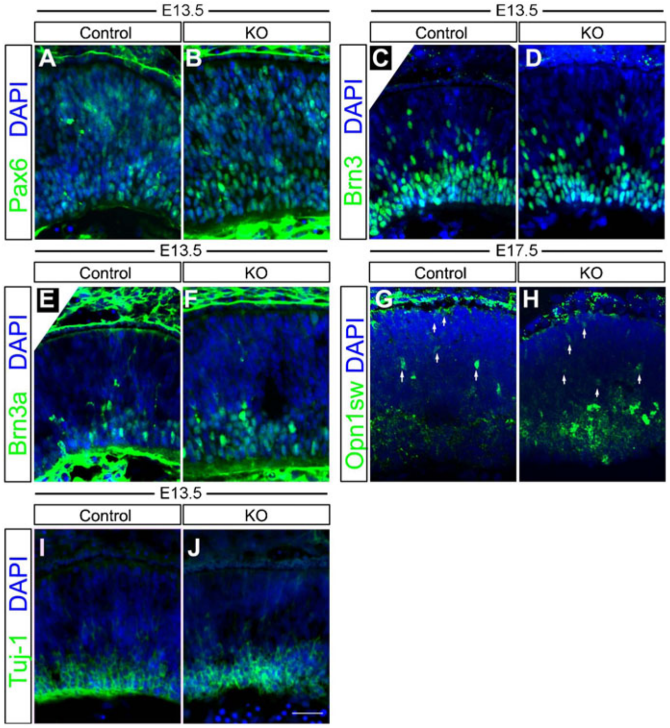

Fig. S3. Top2b deficiency does not affect early retinogenesis. At E13.5, no obvious difference in immunostaining was detected with retinal progenitor marker Pax6 (A,B), ganglion cell marker Brn3 (C,D) and Brn3a (E,F), cone photoreceptor marker Opn1sw (at E17.5) (G,H, arrows), and early neuronal marker Tuj-1 (I,J). Scale bar: 50 µm.

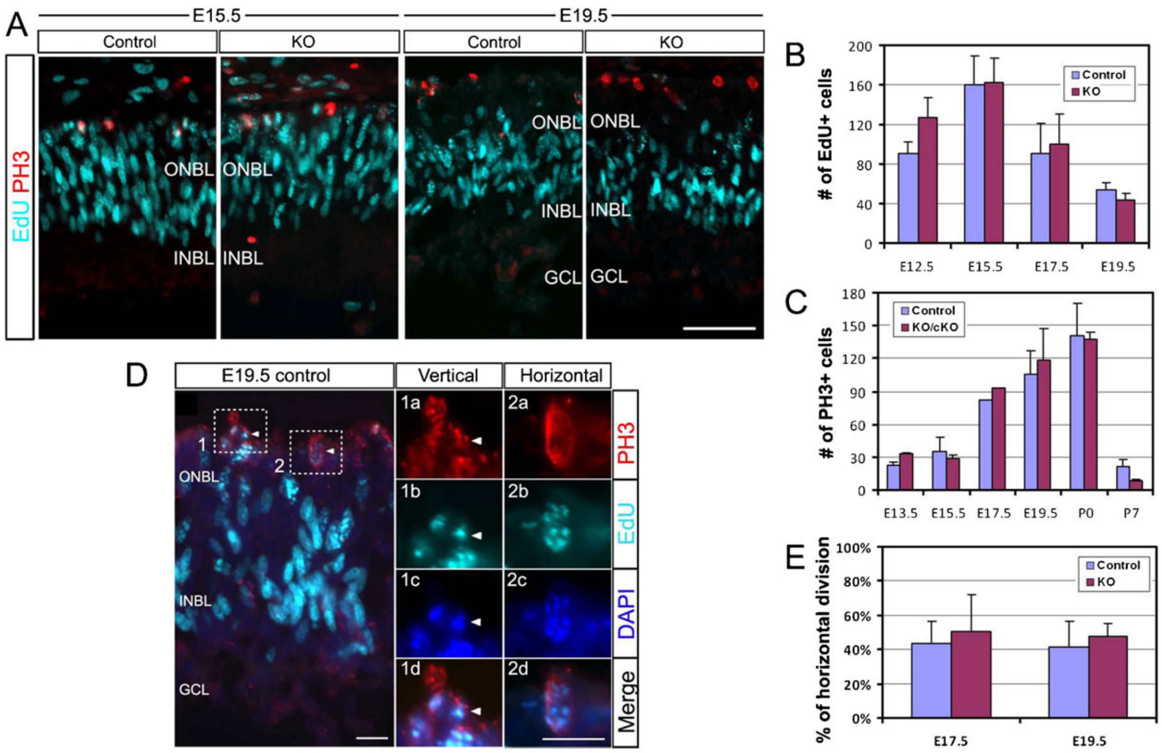

Fig. S4. Top2b deletion does not affect embryonic retinal cell proliferation. The thymidine analog EdU was injected into pregnant female mice 2 hr before sacrifice and dissection. Retina sections were then prepared and EdU incorporation was detected using the Click-iT Edu Alexa Fluor 647 Imaging kit. (A) Cell proliferation in embryonic retina was examined by EdU labeling (S-phase cells, cyan) and phosphorylated-histone 3 (PH3) staining (M-phase cells, red). (B,C) Quantification showed no significant change in the number of EdU+ or PH3+ cells between the control and Top2b-deficient (KO/cKO) retinas. (D) Mitotic cleavage orientation of cell divisions was examined by analyzing EdU+/PH3+ cells. (E) No significant difference in the mitotic cleavage plane was observed. Error bars represent s.d. ($n=3$). INBL, inner neuroblastic layer; ONBL, outer neuroblastic later; GCL, ganglion cell layer. Scale bars: 50 µm.

Biology Open

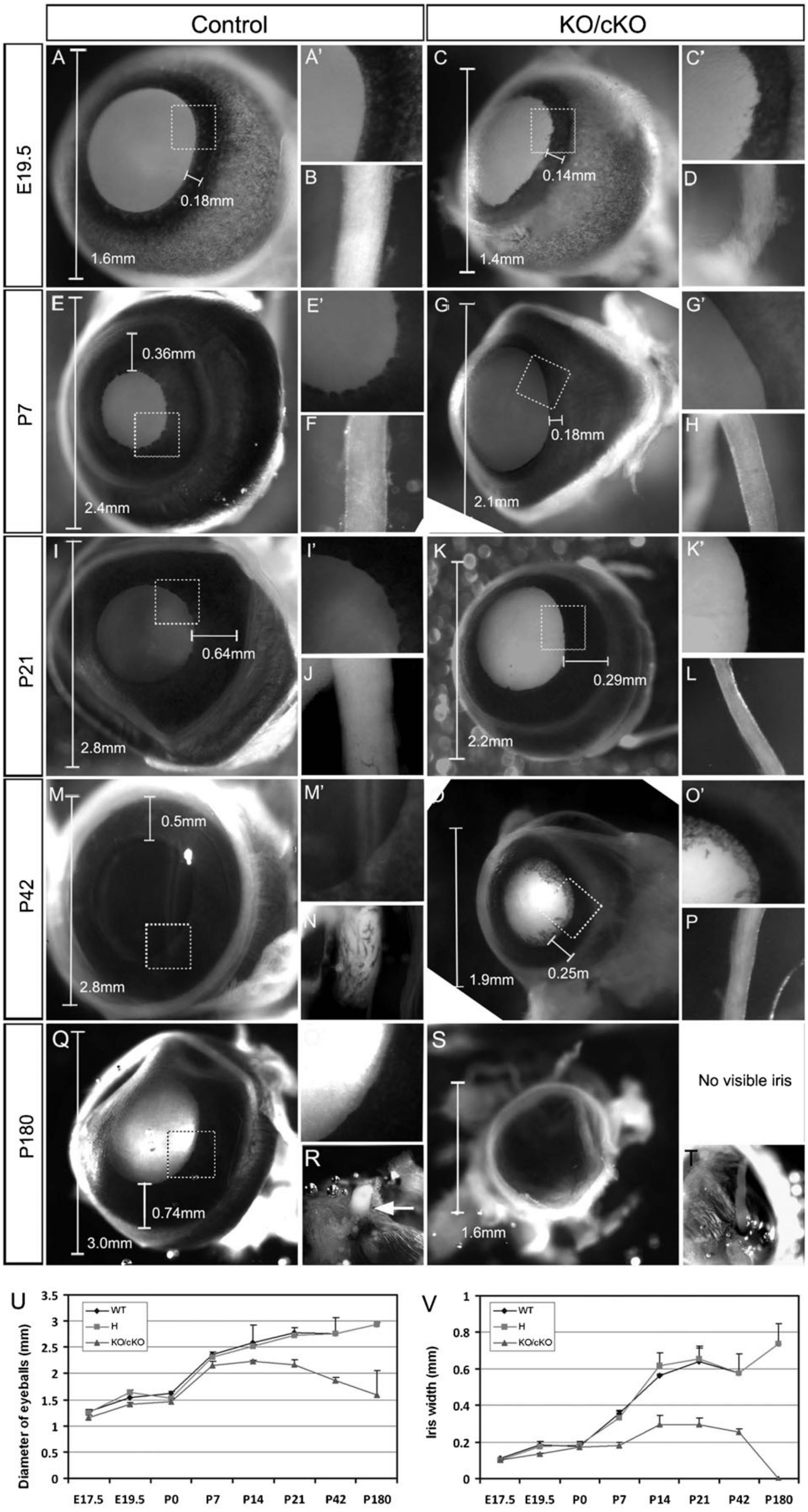

Fig. S5. Top2b deficiency causes morphological changes in the eye. Eyes were isolated from E17.5 and E19.5 wild-type (WT, *Top2b*$^{+/+}$), heterozygotes (H, *Top2b*$^{+/-}$) and KO (*Top2b*$^{-/-}$) embryos; as well as from control (*Top2b*$^{flox2/flox2}$, labeled as WT; H, *DKK3-Cre:Top2b*$^{+/flox2}$) and cKO (*DKK3-Cre:Top2b*$^{flox2/flox2}$) postnatal pups. Diameter of the eye ball and thickness of the iris were measured. (A,C,E,G,I,K,M,O,Q,S) Smaller eyeball, larger pupil and reduced iris thickness were observed in Top2b-deficient eyes. (A′,C′,E′,G′,I′,K′,M′,O′,Q′,S′) Defective iris collarette structure was found in Top2b-deficient eyes. (B,D,F,H,J,L,N,P,R,T) Thinner and flatter optic nerve was observed in Top2b-deficient eyes. (U) Quantification of the diameter of eyeballs. (V) Quantification of the iris width. Error bars represent s.d. (n=3, except n=2 for P0 and P42).

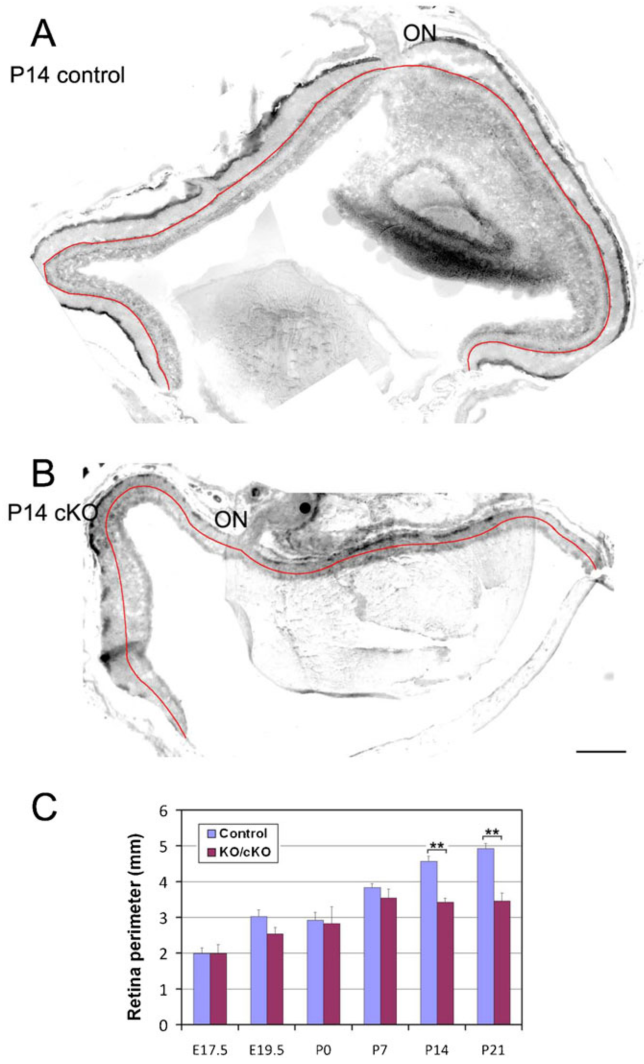

Fig. S6. Reduced perimeter in Top2b-deficient retinas. (A,B) Retina perimeter was determined by measuring the length of the midline (red line) in sections through the central retina where optic nerve (ON) locates.
(C) Quantification showed that retina perimeter was significantly reduced starting at P14 in the Top2b-deficient retinas. Error bars represent s.d. (n=3). Significance was determined by Student's t-test: **p<0.01. Scale bar: 200 µm.

Biology Open

Table S1. A partial list of differentially expressed genes identified by RNA-seq analysis.

		RNA-seq analysis			
Stage	Factor	Log2(fold change)	p-value	Related function	References
P0	Sst*	7.8	3.4E−7	Growth hormone inhibitor; Neurotransmitter; Aid dendrite growth	(Kungel et al., 1997; Bagnoli et al., 2003; Lahlou et al., 2003)
	Igf1	−1.8 E+308	5.8E−12	Mediating cell growth and develop; inducing the differentiation of ganglion cells, rod photoreceptor and one subtype of glial cells	(Meyer-Franke et al., 1995; Fischer et al., 2010; Pinzon-Guzman et al., 2011)
	Hras	−1.9	5.7E−4	Member of Erk pathway, which regulates cell proliferation, differentiation and prevention of apoptosis; member of Igf1 pathway	(Chang et al., 2003)
	Mapk1	−1.2	6.4E−6	Member of Erk pathway, which regulates cell proliferation, differentiation and prevention of apoptosis; member of Igf1 pathway	(Chang et al., 2003)
	HIF1α*	−0.77	5.9E−5	Hif1a pathway; protective function of neurons; member of Igf1 pathway	(Tomita et al., 2003)
	Vegfa	−0.99	1.3E−3	Member of Vegf pathway, which is critical for retinal blood supply; stimulating neurogenesis; member of Igf1 pathway	(Robinson et al., 2001; Jin et al., 2002)
	Rps6b-k1*	−1.6	1.3E−4	Member of Akt pathway; regulating cell growth; member of Igf1 pathway	(Dudek et al., 1997; Harada et al., 2001)
P6	Gfap*	2.2	5.8E−11	Promoting the radial glial cells to re-enter cell cycle	(Guérin et al., 1990; Francke et al., 2001)
	Tac1*	−2.7	5.2E−5	Neurotransmitter; preventing neuronal damage during development and enhancing nerve growth factor-mediated neurite outgrowth	(Bagnoli et al., 2003)
	Grik1*/2	−2.0/−1.7	3.0E−14/1.4E−11	Neurotransmitter receptors	(Dingledine et al., 1999)
	Gria4	−1.3	2.57E−12	Neurotransmitter receptors	(Dingledine et al., 1999)
	Grm7	−1.2	1.2E−4	Neurotransmitter receptors	(Millán et al., 2002)
	Nrxn1/3	−1.2/−2.1	2.0E−6/0	Neurotransmitter receptors; synapse formation	(Uemura et al., 2010)

*indicates the expression of the gene was confirmed with immunohistochemistry analysis (Fig. 7).

Table S2. Differentially expressed genes are involved in neural cell survival and neural system development (Fig. 8).

Stage	Gene sets	Genes
P0	Apoptosis/Programmed cell death	Cdkn1a, Asns, Gadd45a, Dad1, Psen2, Dap3, Pde1b, Dedd, Traf7, Mapk1, Glo1, Raf1, Prkce, Sst, Dap, Cryaa, Apaf1, Tspo, Grin1, Hif1a, VEGFA, Rps6kb1, Atp2c1, Nr3c1, Hras, Gnao1, Gm4617/Ptma, Xrcc6, Srpine2, Ptgd3, Apaf1, Mapk14, Drd2, Igf1, Cdkn1a, Vegfb, Smn1/2, Etv6, Srpk2, P4hb, Creb1
	Cell development	Tspo, Apaf1, Trappc4, Dap, Sst, Nptn, Cryaa, Racgap1, Nlgn1, Raf1, Glo1, Prkce, Mapk1, Asns, Gadd45a, Dad1, Cdkn1a, Nrd1, Traf7, Dedd, Pde1b, Smarca1, Ubb, Thy1, Dap3, Psen2
	Cellular localization	Cdc23, Nup160, Cadps, Pex19, Arfgef2, Kpna4, Kif5a, Vps4b, Atp2c1, Smg7, Pdia3, Syt1, Flna, Nlgn1, Cspg5, Cryaa, Tspo
	Transport	Kcnmb2, Tspo, Slc16a8, Abcg2, Cryaa, Ghrh, Cspg5, Slc11a2, Nlgn1, Flna, Syt1, Pdia3, Smg7, Atp2c1, Necap2, Snap25, Atp11b, Ptgds, Lrp3, Abcd2, Vps4b, Slc25a11, Kif5a, Arfgef2, Kpna4, Nup160, Cadps, Pex19
	Establishment of Cellular localization	Cdc23, Nup160, Cadps, Pex19, Arfgef2, Kpna4, Kif5a, Vps4b, Atp2c1, Smg7, Pdia3, Syt1, Flna, Nlgn1, Cspg5, Cryaa, Tspo
	System Process	Pde6b, Emd, Chrna5, Sst, Cort, Kcnmb2, Drd2, Cryaa, Nptn, Eml2, Atxn7, Gucy1b3, Flii, Kif5a, Kcnip1, nao1, Syt1, Ubb, Nr2e3, Nlgn1, Crx, Mapk1
	Neurological system process	Pde6b, Chrna5, Sst, Cort, Kcnmb2, Drd2, Cryaa, Nptn, Eml2, Nlgn1, Crx, Nr2e3, Atxn7, Syt1, Ubb, Kcnip1, Kif5a, Mapk1
	System Development	Lig1, Dpysl3, Ndufv2, Fabp7, Tpd52, Prps1, Crx, Atp2c1, Adam22, Thy1, Smarca1, Ubb, Ptprz1, Igf1, Nrd1, B3gnt5, Flna, Nlgn1, Racgap1, Emd, Trappc4, Pbx4, Apaf1, Matk, Cspg5, Prdx1, Drd2, Tle1, Nptn, Apba2
	Anatomical structure development	Flna, Nlgn1, Tpd52, Fabp7, Ndufv2, Lig1, Dpysl3, Crx, Prps1, Smarca1, Vcl, Ptprz1, Ubb, Thy1, Atp2c1, Adam22, Igf1, Nrd1, B3gnt5, Apba2, Emd, Racgap1, Trappc4, Prdx1, Pbx4, Apaf1, Kcnmb2, Drd2, Tle1, Cspg5, Dkk3, Matk, Nptn
P6	Neurological system process	Grm7, Syt1, Vsx1, Cartpt, Grik2, Iqcb1, Opn1sw, Grik1, Coch, Nlgn1, Pdc, Cryba1, Rcvrn, Cryba4, Crygd, Cryaa, Crybb3, Cryga, Abca4, Cbln1, Crybb1, Reln, Nrxn1, Gria4, Tac1, GFAP, Qki
	Cytoplasm	Cops5, Chuk, Ergic3, Hspb1, Nos3, Cryaa, Utp11l, Nrsn1, Vdac3, Cct6a, Ubl5, Exoc4, Top2b, Hspe1, Ndufb6, Glo1, Bzw1, Atp5a1, Nefl
